# Tunable switch mediated shikimate biosynthesis in an engineered non-auxotrophic *Escherichia coli*

**DOI:** 10.1038/srep29745

**Published:** 2016-07-13

**Authors:** Pengfei Gu, Tianyuan Su, Qian Wang, Quanfeng Liang, Qingsheng Qi

**Affiliations:** 1 State Key Laboratory of Microbial Technology, Shandong University, Jinan 250100, People’s Republic of China

## Abstract

Shikimate is a key intermediate in the synthesis of neuraminidase inhibitors. Compared with traditional methods, microbial production of shikimate has the advantages of environmental friendliness, low cost, feed stock renewability, and product selectivity and diversity. Despite these advantages, shikimate kinase I and II respectively encoded by *aroK* and *aroL* are inactivated in most shikimate microbial producers, thus requiring the addition of aromatic compounds during the fermentation process. To overcome this problem, we constructed a non-auxotrophic, shikimate-synthesising strain of *Escherichia coli*. By inactivation of repressor proteins, blocking of competitive pathways and overexpression of key enzymes, we increased the shikimate production of wild-type *E. coli* BW25113 to 1.73 g/L. We then designed a tunable switch that can conditionally decrease gene expression and substituted it for the original *aroK* promoters. Expression of *aroK* in the resulting P-9 strain was maintained at a high level during the growth phase and then reduced at a suitable time by addition of an optimal concentration of inducer. In 5-L fed-batch fermentation, strain P-9 produced 13.15 g/L shikimate without the addition of any aromatic compounds. The tunable switch developed in this study is an efficient tool for regulating indispensable genes involved in critical metabolic pathways.

Shikimate, an important intermediate in the aromatic amino acid pathway, can be used as a chiral template for the synthesis of neuraminidase inhibitors such as oseltamivir (Tamiflu^®^)^1^. Shikimate is also a promising building block for the synthesis of other biological compounds^2^,^3^. Because chemical synthetic strategies are restricted by environmental concerns, commercial shikimate production currently involves extraction from seeds of *Illicium* plants-a multistep, low-yield and costly process. To overcome these drawbacks and meet rapidly growing demand, microbial production of shikimate from renewable carbon sources has attracted increasing attention, with several microbial production strains with relatively high titre and yield having been engineered^4^,^5^. *Escherichia coli*, a widely used host for which engineering tools are readily available, is characterised by a clear genetic background and fast growth in inexpensive media and is thus a preferred candidate for shikimate production^6^,^7^,^8^.

In *E. coli*, the first step of the shikimate pathway is the condensation of phosphoenol pyruvate (PEP) and erythrose 4-phosphate (E4P) into 3-deoxy-D-arabinoheptulosonate 7-phosphate (DAHP). This conversion is carried out by DAHP synthase isoenzymes encoded by *aroF*, *aroG* and *aroH*, which are feedback-resistant by L-tyrosine, L-phenylalanine and L-tryptophan, respectively^9^. DAHP is then converted to shikimate via three steps catalysed by dehydroquinate synthase, 3-dehydroquinate dehydratase and shikimate dehydrogenase, respectively. Afterwards, shikimate is transformed to chorismate, a common precursor to L-phenylalanine, L-tyrosine and L-tryptophan (Fig. 1).

Metabolic engineering approaches used to construct a shikimate-producing strain mainly focus on the central carbon metabolic pathway and the shikimate pathway. Inactivation of the PEP:carbohydrate phosphotransferase (PTS) system and overexpression of *tktA* and *ppsA* encoding transketolase and PEP synthase, respectively, are used to increase intracellular levels of E4P and PEP, the two precursors of shikimate^10^,^11^,^12^. These modifications are commonly combined with the plasmid-based overexpression of *aroG*^*FBR*^, *aroB*, *aroD* and *aroE*, which are crucial genes involved in the shikimate synthetic pathway^2^,^6^. To block carbon flux from shikimate into the aromatic amino acid pathway, *aroK* and *aroL* genes encoding shikimate kinase I and II are usually deleted. Recombinant *E. coli* strains with these genetic modifications can produce 0.27 mol shikimate per mol glucose in fed-batch fermentation^13^. To avoid the genetic instability of plasmids, a modified chemically induced chromosomal evolution method has been applied to integrate multiple copies of a gene cluster containing *aroG*^*FBR*^, *aroB*, *aroE* and *tktA* into the *E. coli* chromosome^8^. The final strain, *E. coli* SA116, can produce 3.12 g/L of shikimate, with a yield of 0.33 mol/mol glucose.

Because of the permanent deletion of shikimate kinase encoded by *aroK* and *aroL*, these engineered shikimate producers are auxotrophic. During fermentation, three aromatic amino acids and other aromatic compounds, such as *p*-hydroxybenzoic acid, potassium *p*-aminobenzoate and 2,3-dihydroxybenzoic acid, must therefore be added to the medium to maintain normal host growth, thus increasing the cost of industrial production^14^.

To overcome this problem, in this study we developed an engineered tunable switch that was applied to the controllable expression of *aroK*. In the recombinant strain, the expression of *aroK* was conditionally repressed after accumulation of adequate biomass, thus demonstrating the successful generation of a non-auxotrophic, shikimate-synthesising *E. coli*.

## Results and Discussion

### Construction and characterization of a tunable switch

**Figure 2:**
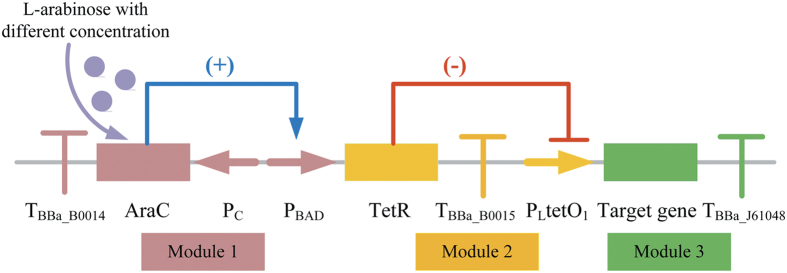
Design of the tunable switch. To simplify the overall construction process, design of the switch was divided into three modules indicated by different colours.

Because the activity of shikimate kinase I encoded by *aroK* is crucial for increasing shikimate accumulation and maintaining normal cell growth, a tunable switch was first constructed to allow appropriate regulation of AroK (Fig. 2). When no inducer was incorporated into the culture, the expression of TetR controlled by P_BAD_ promoter was repressed, with consequent normal expression of the target gene under the regulation of P_LtetO1_. When L-arabinose was added to induce the P_BAD_ promoter, P_LtetO1_ promoter transcription was partially repressed by expressed TetR as a function of L-arabinose concentration, thereby leading to decreased target gene expression.

**Figure 3:**
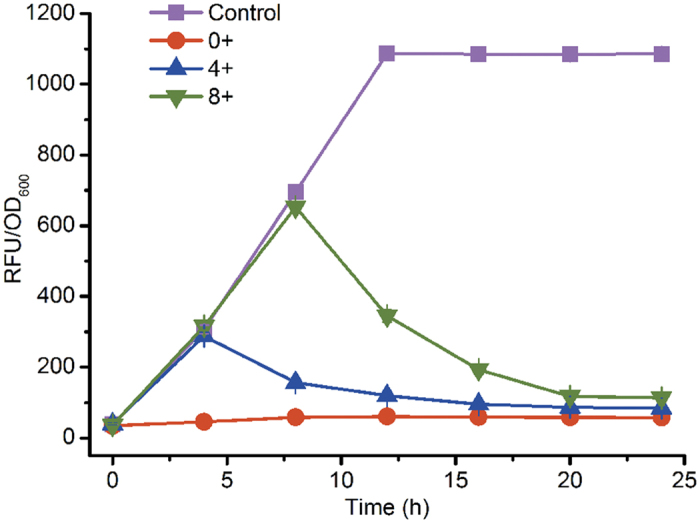
Characterisation of the tunable switch at different supplementation time points. Numbers 0+, 4+ and 8+ indicate strains after supplementation of the medium with 1.00 g/L L-arabinose at 0, 4 or 8 h, respectively, after inoculation of seed cultures. *Escherichia coli* strains were cultured in 50 mL Luria-Bertani medium shaken at 250 rpm and 37 °C. Error bars represent standard deviations based on three replicate fermentations.

To verify the function of this tunable switch, *sfgfp* encoding super-folding green fluorescent protein (sfGFP) was selected as a reporter. When 1.00 g/L L-arabinose was incorporated into the medium at 0, 4, or 8 h after inoculation of seed cultures, the relative fluorescence intensities of sfGFP were all obviously decreased compared with the control strain lacking inducer (Fig. 3). When L-arabinose was added at the start of batch cultivation, fluorescence intensities were maintained at a relatively low level. At 24 h, the fluorescence intensity of strain 0+ was 17.83-fold lower than the control. Similarly, 11.82- and 8.63-fold reductions were observed when supplementation with L-arabinose was delayed for 4 or 8 h, respectively. For strains 4+ and 8+, a delay of approximately 8–12 h occurred before fluorescence intensities reached a relatively low level. Although only a small amount of new sfGFP was generated after repression of the tunable switch, time was still required for proteolysis of existing intracellular sfGFP^15^.

**Figure 4:**
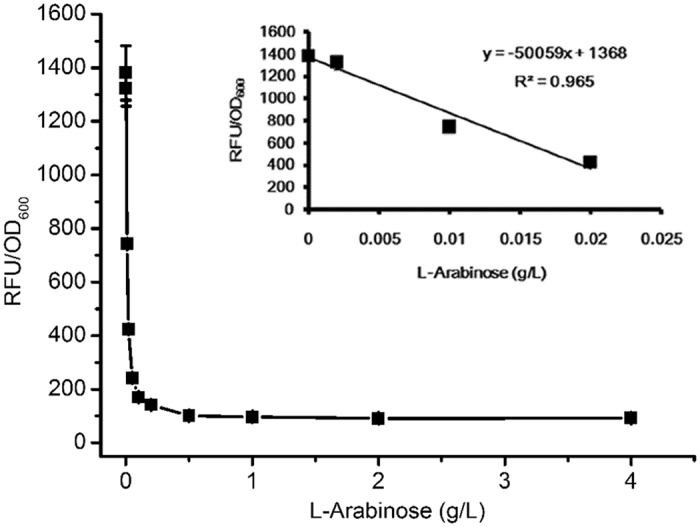
Relationship between concentration of the inducer L-arabinose and repression level of the tunable switch. *Escherichia coli* strains were cultured in 50 mL Luria-Bertani medium shaken at 250 rpm and 37 °C. L-arabinose was added at 0 h and the fluorescence was determined at 12 h. Error bars represent standard deviations based on three replicate fermentations.

We next investigated the relationship between inducer concentration and repression level of the tunable switch. As shown in Fig. 4, relative fluorescence intensity decreased from 1381.53 to 91.52 RFU/OD_600_ when the concentration of L-arabinose was increased from 0 to 2.00 g/L. Increasing the L-arabinose concentration to 4.00 g/L did not further reduce the fluorescence intensity. Within the range of the 0–0.02 g/L inducer, an approximate linear relationship was exhibited between the repression level of the tunable switch and L-arabinose concentration. Even at the highest repression level, achieved by the addition of 2.00 g/L L-arabinose, a fluorescence intensity of 91.53 RFU/OD_600_ was still maintained. Rogers *et al.* have demonstrated that AraC- and TetR-inducible systems exhibit uninduced accumulation of GFP when high-copy vectors are employed^16^. Their results suggest that a small amount of sfGFP can be expressed under the control of P_LtetO1_ even in the presence of the repressor TetR.

### Construction of a shikimate biosynthetic pathway in *E. coli.*

Because shikimate is an important intermediate in the aromatic amino acid pathway, only 1.35 ± 0.13 mg/L shikimate was accumulated during batch fermentation of wild-type *E. coli* BW25113 (Table 1). Metabolic engineering strategies were therefore applied to increase shikimate accumulation. To avoid possible interference, AraC was deleted from the BW25113 genome. To decrease the secretion of acetate, the main by-product in most shikimate-producing strains, *pta* encoding the phosphate acetyltransferase was inactivated. In strain P-2, however, shikimate production was still low (2.25 ± 0.15 mg/L). As has been demonstrated by a carbon flux distribution analysis in wild-type *E. coli*, the PTS system is the largest consumer of PEP, with the relative carbon flux directed to the shikimate pathway representing only 1.5% of consumed PEP^17^. To provide more PEP for shikimate synthesis, we therefore inactivated *ptsG* encoding the IIBC component of the glucose-specific PTS system. As shown in Table 1, strain P-3 achieved a shikimate production of 62.41 mg/L, indicating that the intracellular level of PEP is essential to this process. Because *aroK* encoding shikimate kinase I was selected as the target for the tunable switch, *aroL* encoding shikimate kinase II was deleted to clearly characterise the function of this tunable switch. Compared with P-3, the shikimate production of P-4 increased by 23.71%.

TrpR was next inactivated to eliminate the transcription regulation of genes involved in the shikimate pathway^18^,^19^, which further increased the production of shikimate to 105.03 mg/L (Table 1). In *E. coli*, pyruvate kinase isoenzymes encoded by *pykF* and *pykA* genes can catalyse the formation of pyruvate and Mg-ATP from PEP and Mg-ADP in the presence of potassium^6^. Of these two pyruvate kinase isoenzymes, PykF contributes the most pyruvate kinase activity, and knock out of *pykF* has been reported to improve the production yield of shikimate^20^,^21^. As indicated in Table 1, P-6 with a deletion of *pykF* produced 132.04 mg/L shikimate. Surprisingly, the growth of P-6, as indicated by biomass and the specific growth rate, was recovered compared with that of P-5. Co-inactivation of PykF and the PTS system in *E. coli*, which has been reported to increase carbon flux from PEP into the TCA cycle through oxaloacetate^22^, may be responsible for the comparable growth of P-6 and wild-type BW25113. In addition, the genetic modification from BW25113 to P-6 was accompanied by a 9.25–0.10 g/L decrease in the secretion of acetate.

**Figure 5:**
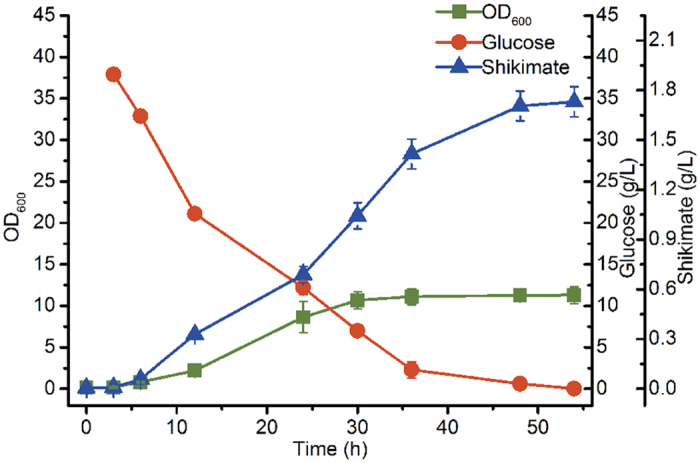
Batch cultivation of *Escherichia coli* P-8 in a 300-mL shake flask. Error bars represent standard deviations based on three replicate fermentations.

To further increase shikimate production and enhance E4P intracellular levels, *aroG*^*FBR*^ and *tktA*, respectively encoding feedback-resistant 3-deoxy-D-arabinoheptulosonate-7-phosphate synthase and transketolase, were co-overexpressed in a low-copy plasmid, pCL1920. In addition, *aroE*, *aroD* and *aroB*, encoding dehydroshikimate reductase, 3-dehydroquinate dehydratase and 3-dehydroquinate synthase separately, were overexpressed in pUC19, which was compatible with pCL1920. Two recombinant plasmids, pF-1 and pF-12, were then simultaneously transformed into strain P-6 to generate P-8. In batch cultivation, strain P-8 exhibited tardy growth for 12 h and then entered into the logarithmic phase (Fig. 5). This long lag phase was possibly owing to the metabolic burden generated by the two recombinant plasmids. After 54 h batch cultivation, P-8 produced 1.73 g/L shikimate, which was 13.11-fold higher than that of P-6, thus indicating that overexpression of the key enzymes in the shikimate pathway is necessary for shikimate production in *E. coli*.

### Conditional repression of AroK with the tunable switch.

**Figure 6:**
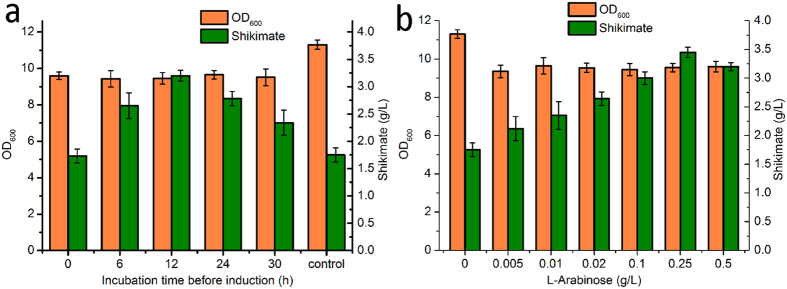
Exploration of the optimal supplementation time point and concentration of L-arabinose for shikimate production. (**a**) The optimal time point for supplementation with L-arabinose. The control strain was P-9 without L-arabinose. (**b**) The optimal supplemental concentration of L-arabinose. Error bars represent standard deviations based on three replicate experiments. *Escherichia coli* strains were cultured in 50 mL fermentation medium shaken at 250 rpm and 37 °C, and shikimate production was determined at 54 h.

By Red recombination, the tunable switch was integrated into the chromosome of P-6 to replace the original promoters, P_aroK1_ and P_aroK2_. As a result, recombinant strain P-7 was obtained, which was then transformed with plasmids pF-1 and pF-12 to generate P-9. To explore the optimal time point for supplementation with inducer, L-arabinose was variously added to the medium at 0, 6, 12, 24 and 30 h. Compared with the P-9 strain without L-arabinose, the other five P-9 strains with different supplemental time points of L-arabinose all exhibited slightly poorer growth (Fig. 6a), possibly because of decreased carbon flow from the downstream aromatic amino acid pathway. After supplementation with L-arabinose, however, higher shikimate accumulation was also observed. In particular, the strains supplemented with L-arabinose at 12 h exhibited the highest shikimate production, 3.22 g/L. In addition, only a slight increase was observed in shikimate production when L-arabinose was added at 0 h, probably the result of the low activity of AroK at the early phase. We next investigated the optimal supplemental concentration of L-arabinose. Seven concentrations ranging from 0 to 0.5 g L^**−**1^ were selected and added to the medium at 12 h during batch fermentation of P-9 (Fig. 6b). Shikimate production increased from 1.75 to 3.45 g/L as the concentration of L-arabinose was increased from 0 to 0.25 g/L. When the concentration of L-arabinose was further increased to 0.5 g/L, however, a slight reduction in the shikimate titre was recorded. Because shikimate production was affected by many factors, quantitative real-time reverse transcription PCR (qRT-PCR) was carried out to directly determine the relationship between the transcription level of *aroK* and the concentration of supplemental L-arabinose. Compared with P-8 lacking the tunable switch, the relative *aroK* transcription of P-9 decreased from 1.05 to 0.09 as the concentration of L-arabinose was increased from 0 to 2.00 g/L (Supplementary Fig. S1). This result was consistent with the relative fluorescence intensity shown in Fig. 4, thus demonstrating that the tunable switch was relatively stable when applied to different target genes.

In previously engineered shikimate-producing strains, *aroK* and *aroL* were permanently knocked out to block the aromatic amino acid biosynthetic pathway. Combined with other genetic modifications, high shikimate accumulation could thereby be achieved, but supplementation with various aromatic compounds was needed to maintain normal growth of the host strain^6^,^7^,^23^. Conditional interruption approaches that can maintain high expression of these genes until adequate cell mass is accumulated are consequently a promising strategy. In this study, we also constructed a recombinant strain, P-11, with an *aroK* deletion. In batch cultivation, P-11 exhibited normal growth and glucose consumption in medium containing aromatic compounds (Supplementary Fig. S2). After 54 h, this strain produced 3.30 g/L shikimate. As the *aroK* gene was deleted in P-11, the downstream pathway of shikimate was completely blocked, which may result in only a slight increasing of shikimate production for P-9 compared to that of P-11. However, supplement of aromatic compounds was indispensable for the culture of P-11, which indicated decreasing the expression level of *aroK* by the tunable switch was more effective and cost-saving than direct deletion of *aroK* in *E. coli* shikimate biosynthesis.

In 2014, Soma *et al.* constructed a metabolic toggle switch to regulate citrate synthase in *E. coli*. By switching *gltA* off and blocking the TCA cycle, the isopropanol production titre and yield were improved up to 3.7 and 3.1 times, respectively^24^. Using this strategy, the same group individually performed conditional repression of *glpK*, *tpiA* and *gapA*, which are involved in glycerol metabolism, and improved the production titre and yield of 3-hydroxypropionic acid by 80 and 94%, respectively^25^. Compared with the complete pathway shutdown accomplished using their metabolic toggle switch, the tunable switch in this study can repress a target gene at different levels. Although no extra aromatic amino acids were added to the medium, strain P-9 exhibited no significant growth defects.

Finally, we compared the by-products of P-8 and P-9 generated in batch fermentation. As shown in Supplementary Fig. S3, the acetate, lactate and pyruvate secreted by P-8 and P-9 were similar. After the introduction of the tunable switch, *aroK* transcription decreased, which led to the lower accumulation of three aromatic amino acids of P-9 compared with P-8. Moreover, strain P-9 exhibited higher quinate production than P-8. In *E. coli*, quinate is generated from 3-dehyroquinic acid catalysed by shikimate dehydrogenase isozymes YdiB and AroE. These two enzymes can also mediate the conversion of 3-dehydroshikimate to shikimate. As the reaction from 3-dehyroquinic acid to shikimate is reversible, the partial blockage of the downstream pathway and accumulation of shikimate in P-9 may have led to alteration of the reaction equilibrium. As a result, more quinate was secreted by P-9^26^,^27^.

### Fermentation of strain P-9 in a 5-L fermentor

**Figure 7:**
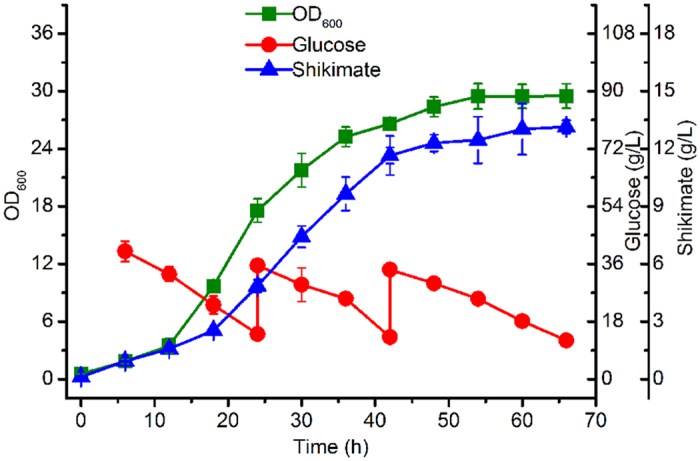
Fed-batch fermentation of P-9 in a 5-L fermentor. Error bars represent standard deviations based on three measurements.

To evaluate the shikimate production potential of strain P-9, fed-batch fermentation was performed in a 5-L fermentor (Fig. 7). This strain exhibited a 12-h lag growth phase. During this period, shikimate also accumulated at low levels. Cell growth then entered the exponential phase. In the middle of the exponential phase, 0.25 g/L L-arabinose was added. After 18 h, the production of shikimate began to increase rapidly. In the P-9 strain, shikimate accumulation was growth dependent. The maximum shikimate accumulation, 13.15 g/L, was achieved at the maximum OD_600_ (29.52). Most importantly, P-9 is a non-auxotrophic shikimate-synthesising strain, with no aromatic compounds needed, thus providing a new avenue for the construction of a promising, economical, industrial shikimate-producing strain.

Other genetic strategies besides metabolic toggle switches have been used to manipulate protein expression levels to balance metabolic fluxes or down-regulate essential genes where a knock-out approach is not applicable. For example, an artificial noncoding small RNA (sRNA) library has been used to isolate and characterise recombinant *E. coli* strains with different repression levels of the endogenous genes *ompF* and *fliC*^28^. The clustered regularly interspaced short palindromic repeats (CRISPR)/Cas system is a powerful tool that has also been shown to perform highly selective transcriptional modulation. By using single-guide RNA targeting different regions of target genes, various repression levels can be achieved^29^. In *E. coli*, the SsrA tag is an 11-amino acid peptide that can render the target protein susceptible to tail-specific proteases^30^. By adding an SsrA tag to the C terminus of TyrA, Doroshenko *et al.* constructed an L-phenylalanine-producing tyrosine-prototrophic *E. coli* strain with unstable TyrA. In M9 medium, this strain exhibited higher L-phenylalanine accumulation and a 10-fold lower Tyr/Phe ratio than the control strain with wild-type TyrA^31^. To ensure proper use of cellular resources, however, the expression of genes encoding enzymes at specific time points must also be controlled to the appropriate level. Compared with the aforementioned strategies, our tunable switch can achieve different repression levels via the direct addition of inducer with different concentrations at different times.

## Methods

### Bacterial strains

All strains, plasmids and primers used in this study are listed in Supplementary Tables S1–S3, respectively. *Escherichia coli* DH5α was used as the host for recombinant DNA manipulation, and *E. coli* BW25113 was used to construct a basic shikimate-producing strain.

### Gene deletion

The *araC* gene encoding an arabinose operon regulatory protein was deleted by the traditional one-step inactivation method^32^. Primers araC-F/araC-R and template plasmid pKD4 were used to obtain the linearised DNA. Primers araC-JF/araC-JR were then selected to verify the positive clones. After generation of strain P-1, the following genes were deleted in turn by the optimised one-step inactivation method^33^: *pta*, *ptsG*, *aroL*, *trpR* and *pykF*, respectively encoding phosphate acetyltransferase, fused phosphoenolpyruvate: carbohydrate phosphotransferase system IIBC components, shikimate kinase II, tryptophan pathway transcriptional repressor and pyruvate kinase I. To construct a control strain, *aroK* was also deleted. Primers pta-F/pta-R, ptsG-F/ptsG-R, aroL-F/aroL-R, trpR-F/trpR-R, pykF-F/pykF-R and aroK-F/aroK-R as well as genomic DNA of strains JW2294-1, JW1087-2, JW0379-1, JW4356-2, JW1666-3 and JW5947-1 were used to generate linearised DNA fragments with extended homologous sequences; in this way, recombinant strains P-2, P-3, P-4, P-5, P-6 and P-10 were successively obtained.

### Plasmid construction

To simplify the whole construction process, generation of the tunable switch was divided into three modules. First, we used the genomic DNA of W3110 as a template and primers BAD-F/BAD-R to obtain the wild-type arabinose module containing AraC and P_BAD_. PCR amplification with template plasmid pF-3 and primers T1-F/T1-R was then used to obtain the terminator BBa_B0014 (iGEM, http://parts.igem.org/). These two DNA fragments with 30–50 homologous arms were assembled and inserted into the *Bam*HI site of pUC19 to generate plasmid pF-4 by the enzymatic assembly strategy^34^. To construct module 2 containing regulatory protein TetR, promoter P_LtetO1_ and terminator BBa_B0015 (iGEM), we used template plasmids pwtCas9 and pF-5 and primers tetR-F/tetR-R, ptetO1-F/ptetO1-R and T2-F/T2-R to obtain these three DNA fragments. Next, homologous arms were added to the original *tetR* gene using primers tetR-YF/tetR-R to generate a new fragment, sTetR. sTetR, promoter P_LtetO1_ and terminator BBa_B0015 were then assembled and inserted into the *Bam*HI site of pUC19 to generate plasmid pF-6. Similarly, fragments of sfGFP and terminator BBa_J61048 (iGEM) with homologous arms were obtained by separately using template plasmids pF-7 and pF-8 and primers gfp-FC/gfp-R and T3-F/T3-R. These two fragments were assembled into pUC19 to generate plasmid pF-9. Finally, primers 1-F/1-R, 2-F/2-R and 3-CF/3-R and templates pF-4, pF-6 and pF-9 were used to obtain three modules with homologous arms that were inserted into the *Bam*HI site of pUC19 to construct pF-2 by the enzymatic assembly strategy.

To obtain DNA fragments of *aroE*, *aroD* and *aroB* by PCR, the genome of BW25113 was selected as a template, with aroE-F/aroE-R, aroD-F/aroD-R and aroB-F/aroB-R respectively used as primers. Promoter P_J23100_ (iGEM) was then assembled with *trpE* by PCR using the original *aroE* fragment as a template and aroE-YF/aroE-R as primers. In addition, the fragment of terminator BBa_B0015 with homologous arms was generated with template plasmid pF-5 and primers T2-NF/T2-NR. The construction of pF-1 was carried out in three steps. First, pF-10 was generated by assembling *aroE* and *aroD* into pUC19, and pF-11 was generated by assembling *aroB* and BBa_B0015 into pUC19. Plasmids pF-10 and pF-11 were then used as templates and aroE-YF/ED-R and BT-F/T2-NR as primers to obtain the two fragments, P_J23100_-*aroE*-*aroD* and *aroB-*T_BBa_B0015_ with homologous arms. Finally, these two fragments were assembled into pUC19 to generate pF-1.

The *aroG*^*FBR*^*-tktA* operon was generated using previously constructed pTAT and primers AT-NF/AT-NR. This fragment was inserted into pCL1920 by *Bam*HI/*Xba*I double-digestion and T4 ligase-based ligation to generate plasmid pF-12.

### Replacement of the original *aroK* promoter by the tunable switch

To improve the integration efficiency of the tunable switch, the chloramphenicol-resistance gene was selected as a marker. PCR using Cm-F/Cm-R and SSD-F/SSD-R as primers and plasmids pKD3 and pF-2 as templates was used to generate two respective DNA fragments, *cat* and the tunable switch, with 30–50 arms homologous to each other. These two fragments were assembled into pUC19 to generate pF-13, which then served as a template plasmid with primers TMTS-F/TMTS-R to yield the final integration fragment. Red recombination was applied to accomplish this integration, and positive clones were verified by primers TMTS-JF/TMTS-JR. The resulting strain, P-7, was transformed with pF-1 and pF-12 to generate P-9. As a control, pF-1 and pF-12 were also transformed into P-10 to generate strain P-11.

### qRT-PCR

Total mRNA of samples was extracted with an RNA Simple Total RNA kit (Tiangen, Beijing, China). Reverse transcription was performed using random 6-mer and oligo dT primers with a PrimeScript RT reagent kit (Takara, Dalian, China). The qRT-PCR was carried out with SYBR Premix Ex Taq II (Takara) following the LightCycler 480 RT-PCR System protocol (Roche, Basel, Switzerland). The qRT-PCR analysis was repeated three times for each sample. The qRT-PCR primers are listed in Supplementary Table S3.

### Growth conditions

Strains for cloning and inoculation were grown in Luria-Bertani (LB) medium (10 g/L tryptone, 5 g/L yeast extract, and 10 g/L NaCl) at 37 °C for 8–12 h. Ampicillin (100 mg/L), chloramphenicol (17 mg/L), kanamycin (25 mg/L), spectinomycin (50 mg/L), or tetracycline (20 mg/L) was incorporated into the medium when necessary. SOB medium (20 g/L tryptone, 5 g/L yeast extract, 0.5 g/L NaCl, 2.5 mM KCl, and 10 mM MgCl_2_) was used for gene deletion. Isopropyl β-D-1-thiogalactopyranoside was added at a final concentration of 0.2 mM. The fermentation medium was as described in a previous study with the three aromatic amino acids excluded^6^. For the cultivation of P-11, however, the three aromatic amino acids were included. A single clone was pre-cultured in 5 mL LB medium at 37 °C on a rotary shaker at 250 rpm overnight. One milliliter of overnight cells were inoculated into 50 mL LB medium and cultured for 8–12 h, with 10% (v/v) seed cultures subsequently incubated into 50 mL fermentation medium at 37 °C and 250 rpm. For fed-batch fermentation, a stirred 5-L glass vessel with the BioFlo310 modular fermentor system (New Brunswick Scientific, Edison, NJ, USA) was used. Single clones were pre-cultured in 50 mL LB medium at 37 °C on a rotary shaker at 250 rpm overnight. Five milliliters of overnight cells were inoculated into 200 mL fermentation medium and cultured for 8–12 h; 10% (v/v) seed cultures were then added to the fermentor containing 3.5 L fermentation medium. Samples were taken at 6-h intervals. When the glucose concentration in the medium fell below 10 g/L, the medium was supplemented with a feeding solution containing 500 g/L glucose to reach a final concentration of approximately 30 g/L. The dissolved oxygen concentration was maintained above 30% air saturation, and the pH was kept at 7.0 through NH_4_OH buffering.

### Analytical methods

Cell growth was monitored at OD_600_ with a spectrophotometer (Shimadzu, Kyoto Japan). Glucose concentration was determined with a glucose biosensor (SBA-40C; Biology Institute of Shandong Academy of Sciences, Jinan, China). Shikimate and quinate were quantitatively analysed on a high-performance liquid chromatography (HPLC) system (Shimadzu) equipped with a HPX-87H column (300 mm × 7.8 mm; 9 μm; Bio-Rad, Hercules, CA, USA) maintained at 50 °C. The mobile phase was 5 mM H_2_SO_4_ at a flow rate of 0.6 mL/min. Peaks were detected using a photodiode array detector at 210 nm. For detection of acetate, lactate, and pyruvate, we used an HPLC system with a refractive index detector (RID-10A) (Shimadzu) and a HPX-87H ion exclusion column (Bio-Rad) at 65 °C. The mobile phase was 5 mM H_2_SO_4_ at a flow rate of 0.6 mL/min. Accumulation of L-phenylalanine, L-tyrosine, and L-tryptophan in strains P-8 and P-9 was determined with a Venusil AA analysis kit (Bonna-Agela Technologies, Tianjin, China). The fluorescence of recombinant strains was determined in 96-well microtitre plates as described previously^33^.

## Additional Information

**How to cite this article**: Gu, P. *et al.* Tunable switch mediated shikimate biosynthesis in an engineered non-auxotrophic *Escherichia coli.* *Sci. Rep.* **6**, 29745; doi: 10.1038/srep29745 (2016).

## Supplementary Material

Supplementary Information

## Figures and Tables

**Figure 1:**
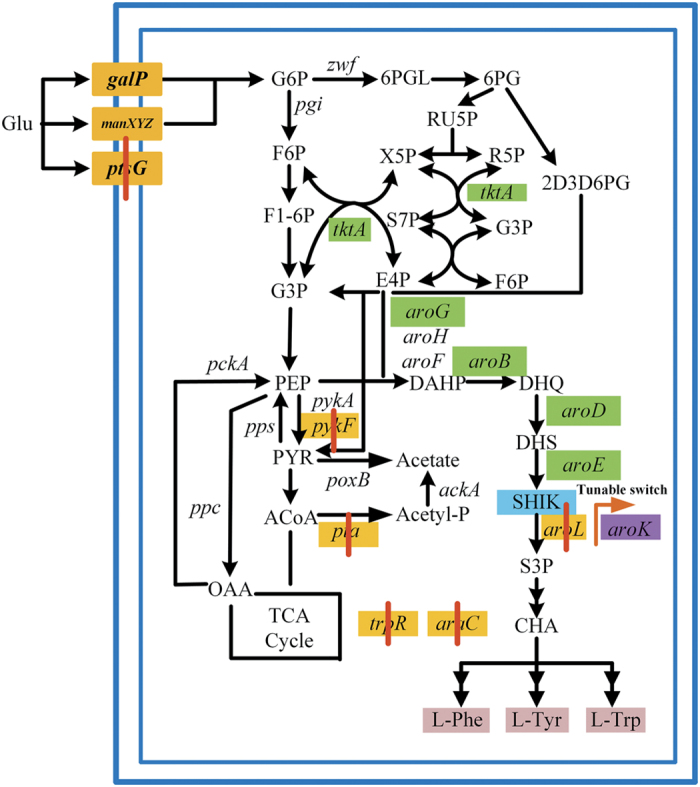
Strategy used to construct the shikimate-synthesising strain P-9. Green boxes represent plasmid-overexpressed genes, and red bars indicate genes that were deleted. The wild-type promoters of *aroK* was replaced by a tunable switch. Glu, glucose; G6P, glucose-6-phosphate; F6P, fructose-6-phosphate; F1-6P, fructose-1, 6-bisphosphate; G3P, glyceraldehyde-3-phosphate; 6PGL,6-phosphoglucono-lactone; 6PG, 6-phosphogluconate; 2D3D6PG, 2-dehydro-3-deoxy-D-gluconate-6-phosphate; RU5P, ribulose-5-phosphate; X5P, xylulose-5-phosphate; R5P, ribose-5-phosphate; S7P, sedoheptulose-7-phosphate; E4P, erythrose-4-phosphate; PEP, phosphoenolpyruvate; PYR, pyruvate; ACoA, acetyl coenzyme A; OAA, oxaloacetic acid; DAHP, 3-deoxy-D-arabino-heptulosonate-7-phosphate; DHQ, 3-dehydroquinate; DHS, 3-dehydroshikimate; SHIK, shikimate; S3P, shikimate-3-phosphate; CHA, chorismate; L-Phe, L-phenylalanine; L-Tyr, L-tyrosine; L-Trp, L-tryptophan.

**Table 1:** Fermentation parameters of strains engineered in shake flasks.

Strains	CDW^b^ (g/L)	Specific growth rate μ (1/h)	Glucose consumption (g/L)	Shikimate (mg/L)	Acetate (g/L)
BW25113	4.25 ± 0.22	0.47 ± 0.05	14.63 ± 0.76	1.35 ± 0.13	9.25 ± 0.37
P-1	3.47 ± ± 0.14	0.43 ± 0.03	14.02 ± 0.43	1.78 ± 0.22	7.16 ± 0.33
P-2	3.60 ± 0.34	0.41 ± 0.05	15.14 ± 0.42	2.25 ± 0.15	5.41 ± 0.35
P-3	3.20 ± 0.21	0.38 ± 0.02	14.82 ± 0.43	62.41 ± 5.97	1.51 ± 0.28
P-4	3.00 ± ± 0.21	0.34 ± 0.04	14.51 ± 0.98	77.21 ± 8.65	0.47 ± 0.08
P-5	2.95 ± 0.21	0.37 ± 0.05	13.60 ± 1.84	105.03 ± 11.32	0.21 ± 0.13
P-6	3.93 ± 0.16	0.51 ± 0.03	9.80 ± 0.76	132.04 ± 9.31	0.10 ± 0.01

^a^Data represent the average of three samples taken from 36-h batch cultures.

^b^Cell dry weight.

## References

[b1] Kim C. U. *et al.* Influenza neuraminidase inhibitors possessing a novel hydrophobic interaction in the enzyme active site: design, synthesis, and structural analysis of carbocyclic sialic acid analogues with potent anti-influenza activity. J Am Chem Soc. 119, 681–690 (1997). 16526129 10.1021/ja963036t

[b2] Kramer M. *et al.* Metabolic engineering for microbial production of shikimic acid. Metab Eng. 5, 277–283 (2003). 14642355 10.1016/j.ymben.2003.09.001

[b3] Adachi O., Ano Y., Toyama H. & Matsushita K. High shikimate production from quinate with two enzymatic systems of acetic acid bacteria. Biosci Biotechnol Biochem. 70, 2579–2582 (2006). 17031026 10.1271/bbb.60259

[b4] Ghosh S. & Banerjee U. C. Generation of *aroE* overexpression mutant of *Bacillus megaterium* for the production of shikimic acid. Microb Cell Fact 14, 69 (2015). 25981549 10.1186/s12934-015-0251-3 PMC4490670

[b5] Liu X., Lin J., Hu H., Zhou B. & Zhu B. Site-specific integration and constitutive expression of key genes into *Escherichia coli* chromosome increases shikimic acid yields. Enzyme Microb Technol. 82, 96–104 (2016). 26672454 10.1016/j.enzmictec.2015.08.018

[b6] Chen X. *et al.* Metabolic engineering of *Escherichia coli* for improving shikimate synthesis from glucose. Bioresour Technol. 166, 64–71 (2014). 24905044 10.1016/j.biortech.2014.05.035

[b7] Escalante A. *et al.* Metabolic engineering for the production of shikimic acid in an evolved *Escherichia coli* strain lacking the phosphoenolpyruvate: carbohydrate phosphotransferase system. Microb Cell Fact. 9, 21 (2010). 20385022 10.1186/1475-2859-9-21 PMC2873404

[b8] Cui Y. Y., Ling C., Zhang Y. Y., Huang J. & Liu J. Z. Production of shikimic acid from *Escherichia coli* through chemically inducible chromosomal evolution and cofactor metabolic engineering. Microb Cell Fact. 13, 21 (2014). 24512078 10.1186/1475-2859-13-21 PMC3923554

[b9] Gu P., Yang F., Kang J., Wang Q. & Qi Q. One-step of tryptophan attenuator inactivation and promoter swapping to improve the production of L-tryptophan in *Escherichia coli*. Microb Cell Fact 11, 30 (2012). 22380540 10.1186/1475-2859-11-30 PMC3311589

[b10] Yi J., Draths K. M., Li K. & Frost J. W. Altered glucose transport and shikimate pathway product yields in *E. coli*. Biotechnol Prog. 19, 1450–1459 (2003). 14524706 10.1021/bp0340584

[b11] Patnaik R., Spitzer R. G. & Liao J. C. Pathway engineering for production of aromatics in *Escherichia coli*: Confirmation of stoichiometric analysis by independent modulation of AroG, TktA, and Pps activities. Biotechnol Bioeng. 46, 361–370 (1995). 18623323 10.1002/bit.260460409

[b12] Lu J. L. & Liao J. C. Metabolic engineering and control analysis for production of aromatics: Role of transaldolase. Biotechnol Bioeng. 53, 132–138 (1997). 18633957 10.1002/(SICI)1097-0290(19970120)53:2<132::AID-BIT2>3.0.CO;2-P

[b13] Chandran S. S. *et al.* Phosphoenolpyruvate availability and the biosynthesis of shikimic acid. Biotechnol Prog. 19, 808–814 (2003). 12790643 10.1021/bp025769p

[b14] Knop D. R. *et al.* Hydroaromatic equilibration during biosynthesis of shikimic acid. J Am Chem Soc. 123, 10173–10182 (2001). 11603966 10.1021/ja0109444

[b15] Fernandez-Castane A., Caminal G. & Lopez-Santin J. Direct measurements of IPTG enable analysis of the induction behavior of *E. coli* in high cell density cultures. Microb Cell Fact. 11, 58 (2012). 22571410 10.1186/1475-2859-11-58 PMC3442970

[b16] Rogers J. K. *et al.* Synthetic biosensors for precise gene control and real-time monitoring of metabolites. Nucleic Acids Res. 43, 7648–7660 (2015). 26152303 10.1093/nar/gkv616 PMC4551912

[b17] Flores S., Gosset G., Flores N., de Graaf A. A. & Bolivar F. Analysis of carbon metabolism in *Escherichia coli* strains with an inactive phosphotransferase system by ^13^C labeling and NMR spectroscopy. Metab Eng. 4, 124–137 (2002). 12009792 10.1006/mben.2001.0209

[b18] Berry A. Improving production of aromatic compounds in *Escherichia coli* by metabolic engineering. Trends Biotechnol. 14, 250–256 (1996). 8771798 10.1016/0167-7799(96)10033-0

[b19] Gunsalus R. P. & Yanofsky C. Nucleotide sequence and expression of *Escherichia coli trpR*, the structural gene for the *trp* aporepressor. Proc Natl Acad Sci USA 77, 7117–7121 (1980). 7012834 10.1073/pnas.77.12.7117 PMC350452

[b20] Rodriguez A. *et al.* Constitutive expression of selected genes from the pentose phosphate and aromatic pathways increases the shikimic acid yield in high-glucose batch cultures of an *Escherichia coli* strain lacking PTS and *pykF*. Microb Cell Fact. 12, 86 (2013). 24079972 10.1186/1475-2859-12-86 PMC3852013

[b21] Licona-Cassani C. *et al.* Inactivation of pyruvate kinase or the phosphoenolpyruvate: sugar phosphotransferase system increases shikimic and dehydroshikimic acid yields from glucose in *Bacillus subtilis*. J Mol Microbiol Biotechnol. 24, 37–45 (2014). 24158146 10.1159/000355264

[b22] Meza E., Becker J., Bolivar F., Gosset G. & Wittmann C. Consequences of phosphoenolpyruvate:sugar phosphotranferase system and pyruvate kinase isozymes inactivation in central carbon metabolism flux distribution in *Escherichia coli*. Microb Cell Fact. 11, 127 (2012). 22973998 10.1186/1475-2859-11-127 PMC3521201

[b23] Ths K. M. & Knop D. R. & Frost, J. W. Shikimic Acid and Quinic Acid: Replacing isolation from plant sources with recombinant microbial biocatalysis. J Am Chem Soc. 121 (1999).

[b24] Soma Y., Tsuruno K., Wada M., Yokota A. & Hanai T. Metabolic flux redirection from a central metabolic pathway toward a synthetic pathway using a metabolic toggle switch. Metab Eng 23, 175–184 (2014). 24576819 10.1016/j.ymben.2014.02.008

[b25] Tsuruno K., Honjo H. & Hanai T. Enhancement of 3-hydroxypropionic acid production from glycerol by using a metabolic toggle switch. Microb Cell Fact. 14, 155 (2015). 26438162 10.1186/s12934-015-0342-1 PMC4594890

[b26] Juminaga D. *et al.* Modular engineering of L-tyrosine production in *Escherichia coli*. Appl Environ Microbiol. 78, 89–98 (2012). 22020510 10.1128/AEM.06017-11 PMC3255607

[b27] Kim B. G., Jung W. D., Mok H. & Ahn J. H. Production of hydroxycinnamoyl-shikimates and chlorogenic acid in *Escherichia coli*: production of hydroxycinnamic acid conjugates. Microb Cell Fact. 12, 15 (2013). 23383718 10.1186/1475-2859-12-15 PMC3621256

[b28] Sharma V., Yamamura A. & Yokobayashi Y. Engineering artificial small RNAs for conditional gene silencing in *Escherichia coli*. ACS Synth Biol. 1, 6–13 (2012). 23651005 10.1021/sb200001q

[b29] Cress B. F. *et al.* CRISPathBrick: Modular combinatorial assembly of type II-A CRISPR arrays for dCas9-mediated multiplex transcriptional repression in *E. coli*. ACS Synth Biol. 4, 987–1000 (2015). 25822415 10.1021/acssynbio.5b00012

[b30] Gottesman S., Roche E., Zhou Y. & Sauer R. T. The ClpXP and ClpAP proteases degrade proteins with carboxy-terminal peptide tails added by the SsrA-tagging system. Genes Dev. 12, 1338–1347 (1998). 9573050 10.1101/gad.12.9.1338 PMC316764

[b31] Doroshenko V. G. *et al.* Construction of an L-phenylalanine-producing tyrosine-prototrophic *Escherichia coli* strain using *tyrA ssrA*-like tagged alleles. Biotechnol Lett. 32, 1117–1121 (2010). 20364292 10.1007/s10529-010-0265-1

[b32] Datsenko K. A. & Wanner B. L. One-step inactivation of chromosomal genes in *Escherichia coli* K-12 using PCR products. Proc Natl Acad Sci USA 97, 6640–6645 (2000). 10829079 10.1073/pnas.120163297 PMC18686

[b33] Li M. *et al.* Extending homologous sequence based on the single gene mutants by one-step PCR for efficient multiple gene knockouts. *Folia Microbiol* (*Praha*) 57, 209–214 (2012). 22461081 10.1007/s12223-012-0111-z

[b34] Gibson D. G. *et al.* Enzymatic assembly of DNA molecules up to several hundred kilobases. Nat Methods 6, 343–345 (2009). 19363495 10.1038/nmeth.1318

[b35] Pedelacq J. D., Cabantous S., Tran T., Terwilliger T. C. & Waldo G. S. Engineering and characterization of a superfolder green fluorescent protein. Nat Biotechnol. 24, 79–88 (2006) 16369541 10.1038/nbt1172

