# Supplementary material for: Tunable switch mediated shikimate biosynthesis in an engineered non-auxotrophic Escherichia coli
Source: Sci Rep. 2016 Jul 13;6:29745. doi: 10.1038/srep29745 (PMC4942831; doi:10.1038/srep29745)
Supplement: Supplementary Information [file srep29745-s1.doc]

**Supplementary Information**

**Tunable switch mediated shikimate biosynthesis in an engineered non-auxotrophic *Escherichia coli***

Pengfei Gu, Tianyuan Su, Qian Wang, Quanfeng Liang^*^, Qingsheng Qi

State Key Laboratory of Microbial Technology, Shandong University, Jinan 250100, People’s Republic of China

* Corresponding author.

Tel.: +86-531-88362897;

Fax: +86-531-88565610;

E-mail: [liangquanfeng@sdu.edu.cn](mailto:liangquanfeng@sdu.edu.cn)

**Supplementary Tables**

**Supplementary Table S1. Strains used in this study**

| Name | Relevant genotype | Reference |
| --- | --- | --- |
| DH5α | *F^-^*, *endA1*, *hsdR17* (*r_K_^-^*, *m_K_^+^*), *supE44*, *thi-l*, *λ^-^*, *recA1*, *gyrA96*, Δ*lacU169* (*Φ80lacZ* Δ*M15*) | Lab stock |
| BW25113 | F-, *Δ(araD-araB)567*, *ΔlacZ4787*(::rrnB-3), *λ^-^*, *rph-1*, *Δ(rhaD-rhaB)568*, *hsdR514* | Lab stock |
| W3110 | F-, *λ^-^*, *IN(rrnD-rrnE)1*, *rph-1* | Lab stock |
| P-1 | BW25113(*ΔaraC*) | This study |
| P-2 | P-1(*Δpta*) | This study |
| P-3 | P-2(*ΔptsG*) | This study |
| P-4 | P-3(*ΔaroL*) | This study |
| P-5 | P-4(*ΔtrpR*) | This study |
| P-6 | P-5(*ΔpykF*) | This study |
| P-7 | P-6 with the wild *aroK* promoter replaced by the tunable switch | This study |
| P-8 | P-6 containing pF-1 and pF-12 | This study |
| P-9 | P-7 containing pF-1 and pF-12 | This study |
| P-10 | P-6 (*ΔaroK*) | This study |
| P-11 | P-10 containing pF-1 and pF-12 | This study |
| JW2294-1 | F-, *Δ(araD-araB)567*, *ΔlacZ4787*(::rrnB-3), *λ^-^*, *Δpta-779::kan*, *rph-1*, *Δ(rhaD-rhaB)568*, *hsdR514* | CGSC |
| JW1087-2 | F-, *Δ(araD-araB)567*, *ΔlacZ4787*(::rrnB-3), *λ^-^*, *ΔptsG763::kan*, *rph-1*, *Δ(rhaD-rhaB)568*, *hsdR514* | CGSC |
| JW0379-1 | F-, *Δ(araD-araB)567*, *ΔlacZ4787*(::rrnB-3), *ΔaroL753::kan*, *λ^-^*, *rph-1*, *Δ(rhaD-rhaB)568*, *hsdR514* | CGSC |
| JW4356-2 | F-, *Δ(araD-araB)567*, *ΔlacZ4787*(::rrnB-3), *λ^-^*, *rph-1*, *Δ(rhaD-rhaB)568*, *hsdR514*, *ΔtrpR789::kan* | CGSC |
| JW1666-3 | F-, *Δ(araD-araB)567*, *ΔlacZ4787*(::rrnB-3), *λ^-^*, *ΔpykF751::kan*, *rph-1*, *Δ(rhaD-rhaB)568*, *hsdR514* | CGSC |
| JW5947-1 | F-, *Δ(araD-araB)567*, *ΔlacZ4787(::rrnB-3)*, *λ-*, *ΔaroK725::kan*, *rph-1*, *Δ(rhaD-rhaB)568*, *hsdR514* | CGSC |

**Supplementary Table S2. Plasmids used in this study**

| Name | Relevant genotype | Reference |
| --- | --- | --- |
| pKD3  pKD4  pKD46 | *bla*, FRT-*cat*-FRT  *bla*, FRT-*kan*-FRT  *bla*, helper plasmid | 30  30  30 |
| pCP20 | *bla* and *cat*, helper plasmid | 36 |
| pCL1920 | Spc^R^, pSC101ori, and *lac* promoter | 37 |
| pUC19 | Amp^R^, pMB1 ori, and *lac* promoter | Lab stock |
| pTAT | pCL1920 containing *aroG^FBR^*,*trpE^FBR^*, and *tktA* | 9 |
| pwtCas9 | aTc-inducible expression of wild-type Cas9, Amp^R^ | 38 |
| pF-1 | pUC19-*aroE*-*aroD*-*aroB*-T2 | This study |
| pF-2 | pUC19 containing the tunable switch and *sfgfp* | This study |
| pF-3 | pUC19-T1 | BGI |
| pF-4 | pUC19-BAD-T1 | This study |
| pF-5 | pUC19-T2 | BGI |
| pF-6 | pUC19-*tetR*-T2-P_LtetO1_ | This study |
| pF-7 | pUC19-*sfgfp* | BGI |
| pF-8 | pUC19-T3 | BGI |
| pF-9 | pUC19-*sfgfp*- T3 | This study |
| pF-10 | pUC19-*aroE*-*aroD* | This study |
| pF-11 | pUC19- *aroB*-T2 | This study |
| pF-12 | pCL1920-*aroG^FBR^-tktA* | This study |
| pF-13 | pUC19 containing the tunable switch and *cat* | This study |

**Supplementary Table S3. Oligonucleotides used in this study**

| Name | Sequence (5’-3’) |
| --- | --- |
| araC-F | TATGGACAATTGGTTTCTTCTCTGAATGGTGGGAGTATGAAAAGTGTGTAGGCTGGAGCTGCTTC |
| araC -R | TATGCTACTCCGTCAAGCCGTCAATTGTCTGATTCGTTACCAAATGGGAATTAGCCATGGTCC |
| araC-JF | ATGGCTTTGGTCCCGCTTTGTTA |
| araC-JR | AGCCAGGCGTGCCAGAAACTT |
| pta-F | GGCATGAGCGTTGACGCAATCAACAAACTGCT |
| pta-R | GATCCTGAGGTTAATCCTTCAAACGGGAAGTTC |
| ptsG-F | ATCGGTTACTGGTGGAAACTGACTCACCTT |
| ptsG-R | GTGGATGGGACAGTCAGTAAAGGGGTGGAA |
| trpR-F | CCGAAAGTGAAATCACCGGTAGGGGCCAGCG |
| trpR-R | TAGCCAATGCCCGCCGTTTACTTCCAGAGG |
| aroL-F | GGCGATTTGGTGATCACCGCAGATATACCTTTGGCT |
| aroL-R | TCGTCGTTAATTACCGGGCAAGTGTGAAGCA |
| pykF-F | ACAAGTTGTATATTTTTTGAAACGCTG |
| pykF-R | TTGAGTTAATCTCCATGTAGCGT |
| aroK-F | TCCCTGGTTCGGGCAATTATTTCGT |
| aroK-R | ATCGCCCACTACGCCGCCGCCAAGC |
| aroK-QF | GCTGATGTGGGCTGGGTTT |
| aroK-QR | TTCAAGATAAACGACAACGC |
| rrsG-QF | GGTGTAGCGGTGAAATGCGTAG |
| rrsG-QR | TCAAGGGCACAACCTCCAAGTC |
| BAD-F | TTATGACAACTTGACGGCTACATCATTCACTTTTTCTTCACAACCGG |
| BAD-R | ACGCCAAGCTTGCATGCCTGCAGGTCGACTCTAGAGGATCACAGTAGAGAGTTGCGATAAA |
| T1-F | AAACGACGGCCAGTGAATTCGAGCTCGGTACCCGGGGATCTCACACTGGCTCACCTTCGGGT |
| T1-R | AAGTGAATGATGTAGCCGTCAAGTTGTCATAAAAATAATAAAAAAGCCGGAT |
| tetR-F | TTCTCCATACCCGTTTTTTTGGGCTAGCTACTAGAGAAAGAGGAGAAAATGATGTCTAGATTAGATAAA |
| tetR-YF | AAACGACGGCCAGTGAATTCGAGCTCGGTACCCGGGGATCTTCTCCATACCCGTTTTTTTGGGCTA |
| tetR-R | TTAAGACCCACTTTCACATTTAAGTTGTTT |
| T2-F | CGGATTAGAAAAACAACTTAAATGTGAAAGTGGGTCTTAACCAGGCATCAAATAAAACGAAAGGCT |
| T2-R | TATAAACGCAGAAAGGCCCACCCGAAGG |
| pteto1-F | CACACTGGCTCACCTTCGGGTGGGCCTTTCTGCGTTTATATCCCTATCAGTGATAGAGATTGA |
| pteto1-R | ACGCCAAGCTTGCATGCCTGCAGGTCGACTCTAGAGGATCGTGCTCAGTATCTCTATCACTGATA |
| gfp-FC | AACGACGGCCAGTGAATTCGAGCTCGGTACCCGGGGATCTACTAGAGAAAGAGGAGAAATACTAG |
| gfp-YF | AACGACGGCCAGTGAATTCGAGCTCGGTACCCGGGGATCTTTACGGCTAGCTCAGCCCT |
| gfp-R | TTTACGTAAATCAGGTGAAACTGACCGATAAGCCGGTTCATCATTTGTACAGTTCATCCATA |
| T3-F | ACCGGCTTATCGGTCAGTTTCACCT |
| T3-R | ACGCCAAGCTTGCATGCCTGCAGGTCGACTCTAGAGGATCTTTCTTTTGGGTATAGCGTCGTGGA |
| 1-F | AAACGACGGCCAGTGAATTCGAGCTCGGTACCCGGGGATCTCACACTGGCTCACCTTCGGGTGGGCCTTT |
| 1-R | ACAGTAGAGAGTTGCGATAAAAAGCGTCAGGTAGG |
| 2-F | GCGGATCCTACCTGACGCTTTTTATCGCAACTCTCTACTGTTTCTCCATACCCGTTTTTTTGGGCTA |
| 2-R | GTGCTCAGTATCTCTATCACTGATAGGGATG |
| 3-07F | AGAGATTGACATCCCTATCAGTGATAGAGATACTGAGCACTTTACGGCTAGCTCAGCCCTAGGTATTATG |
| 3-CF | AGAGATTGACATCCCTATCAGTGATAGAGATACTGAGCACTACTAGAGAAAGAGGAGAAATACTAG |
| 3-R | ACGCCAAGCTTGCATGCCTGCAGGTCGACTCTAGAGGATCTTTCTTTTGGGTATAGCGTCGTGGACAGT |
| aroE-F | TTGACGGCTAGCTCAGTCCTAGGTACAGTGCTAGCTACTAGAGAAAGAGGAGAAATACTAGATGGAAACCTATGCTGT |
| aroE-YF | AAACGACGGCCAGTGAATTCGAGCTCGGTACCCGGGGATCTTGACGGCTAGCTCAGTCCTAGGTAC |
| aroE-R | TCACGCGGACAATTCCTCCTGCAA |
| aroD-F | CCAGTTATAAAGCAATTGCAGGAGGAATTGTCCGCGTGAAAAGAGGAGAAATACTAGATGAAAACCGTAACTGTAAA |
| aroD-R | ACGCCAAGCTTGCATGCCTGCAGGTCGACTCTAGAGGATCTTATGCCTGGTGTAAAATAGTTAAT |
| aroB-F | AAACGACGGCCAGTGAATTCGAGCTCGGTACCCGGGGATCAAAGAGGAGAAATACTAGATGGAGAGGATTGTCGTTAC |
| aroB-R | TTACGCTGATTGACAATCGGCAATGG |
| T2-NF | CTTGTTCTTAACGCCATTGCCGATTGTCAATCAGCGTAACCAGGCATCAAATAAAACGAAAGGC |
| T2-NR | ACGCCAAGCTTGCATGCCTGCAGGTCGACTCTAGAGGATCTATAAACGCAGAAAGGCCCACCCGAAGGT |
| ED-R | TTATGCCTGGTGTAAAATAGTTAAT |
| BT-F | GATTTGCGCACGGTATTAACTATTTTACACCAGGCATAAAAAGAGGAGAAATACTAGATGGAGAGGATT |
| AT-NF | TGCTCTAGAAAAGAGGAGAAATACTAGATGAATTATCAGAACGACGATTTACGCATCAAAGAAATCAAAG |
| AT-NR | CGCGGATCCTTACAGCAGTTCTTTTGCTTTCGCAACAACGTTATCAACAGTG |
| SSD-F | CTAAGGAGGATATTCATATGGACCATGGCTAATTCCCATTCACACTGGCTCACCTTCGGGTGGGCCTTT |
| SSD-R | ACGCCAAGCTTGCATGCCTGCAGGTCGACTCTAGAGGATCCTAGTATTTCTCCTCTTTCTCTAGTA |
| Cm-F | AAACGACGGCCAGTGAATTCGAGCTCGGTACCCGGGGATCGTGTAGGCTGGAGCTGCTTC |
| Cm-R | ATGGGAATTAGCCATGGTCC |
| TMTS-F | CGAGTTAGTGGTGTTTATCACGCCACGACTGGTTTCCAGTGAGTAAGTGTAGGCTGGAGCTGCTTCGAAGTTCCTATAC |
| TMTS-R | GCACCCATAGGCCCAACCAGAAAGATATTGCGTTTCTCTGCCATCTAGTATTTCTCCTCTTTCTCTAGTA |
| TMTS-JF | CAGCAGCTGGATATTATCGCCAGTCCGCGTCTGCT |
| TMTS-JR | ATGAAGCTGGTTCATTAAACAAACCAGATGCGATGGTAATTGGGT |

**Supplementary Figures**

**Supplementary Figure S1.** Effect of L-arabinose concentration on the transcription of *aroK*. *Escherichia coli* strains were cultured in 50 mL fermentation medium shaken at 250 rpm and 37 °C. L-arabinose was added at 12 h. Error bars represent standard deviations based on three replicate fermentations.


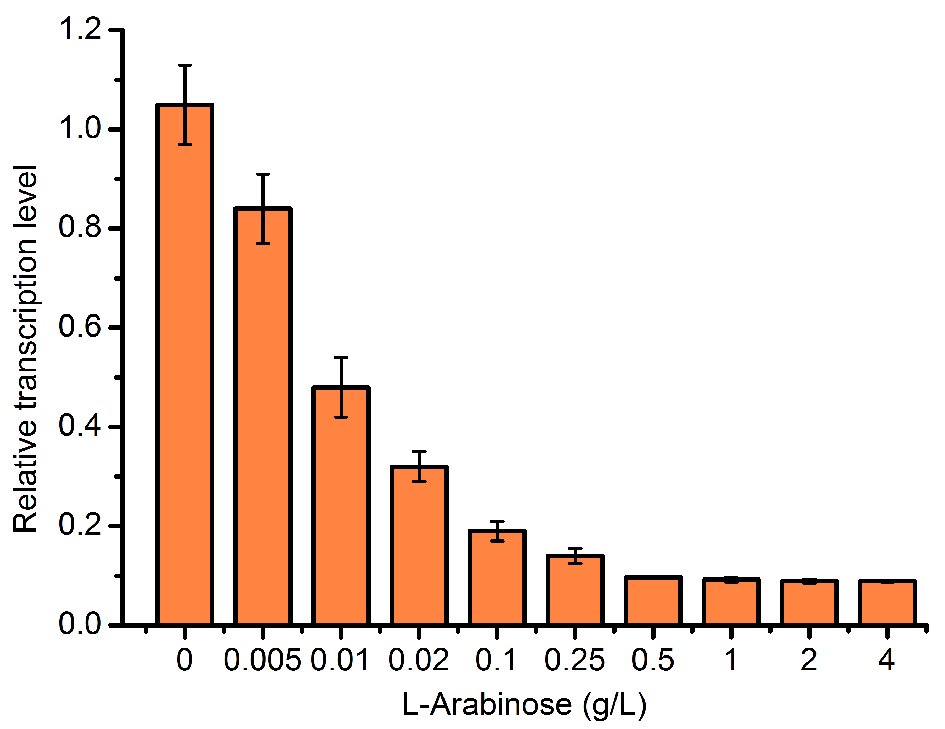


**Supplementary Figure S2.** Batch cultivation of *Escherichia coli* P-11 in 300-mL shake flasks. Error bars represent standard deviations based on three replicate fermentations.


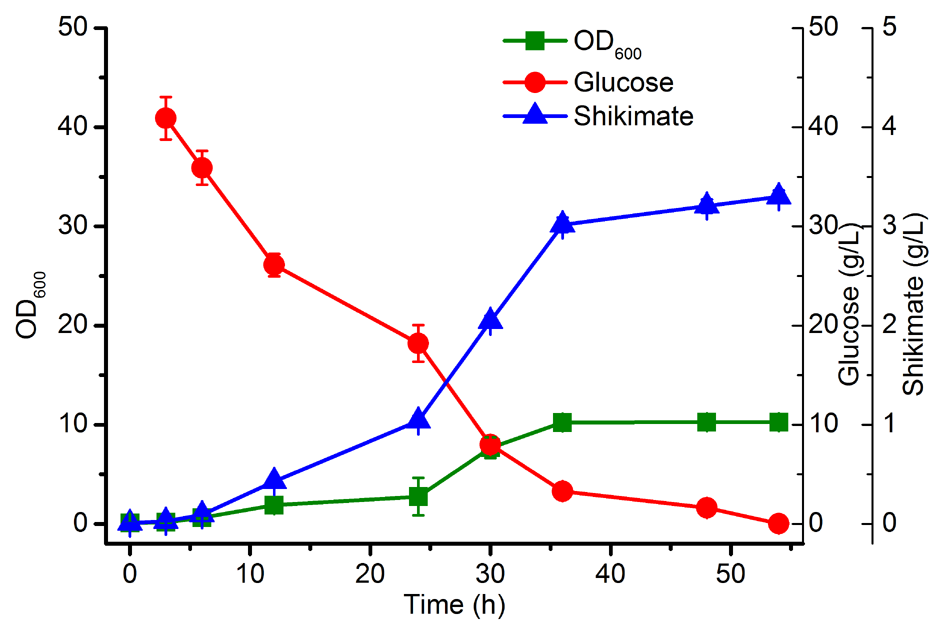


**Supplementary Figure S3.** By-product accumulation of P-8 and P-9 in batch fermentation. *Escherichia coli* strains were cultured in 50 mL fermentation medium shaken at 250 rpm and 37 °C, and shikimate production was determined at 54 h. Error bars represent standard deviations based on three replicate experiments.


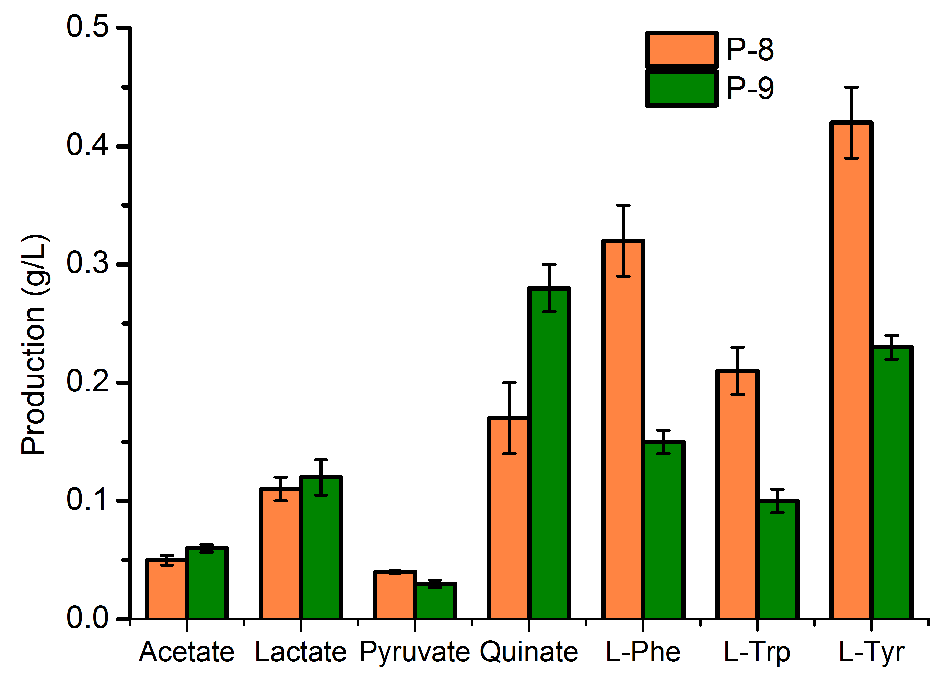


**References**

30 Datsenko, K. A. & Wanner, B. L. One-step inactivation of chromosomal genes in *Escherichia coli* K-12 using PCR products. Proc Natl Acad Sci U S A 97, 6640-6645 (2000).

36 Cherepanov, P. P. & Wackernagel, W. Gene disruption in *Escherichia coli*: TcR and KmR cassettes with the option of Flp-catalyzed excision of the antibiotic-resistance determinant. *Gene* **158**, 9-14 (1995).

37 Lerner, C. G. & Inouye, M. Low copy number plasmids for regulated low-level expression of cloned genes in *Escherichia coli* with blue/white insert screening capability. *Nucleic Acids Res* **18**, 4631 (1990).

9 Gu, P., Yang, F., Kang, J., Wang, Q. & Qi, Q. One-step of tryptophan attenuator inactivation and promoter swapping to improve the production of L-tryptophan in *Escherichia coli*. *Microb Cell Fact* **11**, 30 (2012).

38 Qi, L. S. et al. Repurposing CRISPR as an RNA-guided platform for sequence-specific control of gene expression. Cell 152, 1173-1183 (2013).
